# Editorial: Biotechnological Potential of Plant-Microbe Interactions in Environmental Decontamination

**DOI:** 10.3389/fpls.2019.01519

**Published:** 2019-11-26

**Authors:** Ying Ma

**Affiliations:** College of Resources and Environment, Southwest University, Chongqing, China

**Keywords:** plant growth promoting microorganisms, plant-microbe-metal interactions, biotechnology, climate change induced-stresses, environmental decontamination

Soil contamination with heavy metals and organic contaminants has become a major global environmental problem. Phytoremediation, use of plants to immobilize, extract metals or degrade organic pollutants, provides a cost-effective eco-benign alternative to traditional methods. In most cases, plants act indirectly by stimulating beneficial rhizosphere and endophytic microbes, which could facilitate/accelerate phytoremediation process by improving plant growth, altering soil metal bioavailability or facilitating the degradation of organic pollutants (known as bioaugmentation). Plant growth-promoting microorganisms (PGPM) [e.g., plant growth-promoting bacteria (PGPB), rhizobia, and arbuscular mycorrhizal fungi (AMF)] exhibiting plant growth-promoting (PGP) traits [e.g., synthesis of indole-3-acetic acid (IAA), 1-aminocyclopropane-1-carboxylate (ACC) deaminase, siderophores, surfactants, nitrogen (N) fixation, solubilization of phosphate (P) and potassium (K)] can enhance plant biomass production. Furthermore, AMF can contribute considerably to the short-term underground carbon (C) sequestration by retaining photosynthate C transferred by their host plant and/or stabilizing soil aggregate in the phytoremediation systems. In the case of organic pollutants, the application of pollutant-degrading bacteria and fungi can improve phytoremediation due to their ability to partially degrade organic pollutants or metabolize pollutant degradation products to CO_2_ and water. Regarding heavy metal decontamination, the release of organic acids and acidification of rhizosphere soils by metal-mobilizing microbes may facilitate phytoextraction, whereas the release of root exudates (such as sugars, amino acids, and enzymes) and precipitation of metal-immobilizing bacteria are beneficial to phytostabilization. However, the knowledge on the understanding of soil microbial communities and their functions, plant performance and metabolism, as well as environmental conditions that promote predictable activities of both plants and microbes in polluted soils is far from complete. Therefore, this research topic was launched to advance our knowledge of integrated response of plant-microbe-soil interactions and underlying mechanisms, and review recent progress on how the estimable environmental microbial biotechnologies can be used to boost plant growth and metabolism, appropriate microbial community assembly, and eventually improve phytoremediation efficiency, therefore contributing to translate basic knowledge into sustainable applications.

Plant-microbe interactions play a critical role in plant adaption to metalliferous environments, stimulation of plant growth, and thus can be explored to accelerate microbe-aided phytoremediation. Ma et al. (2016a) extensively reviewed the recent advances to understand the biochemical (e.g., chemotaxis, colonization, beneficial functioning) and molecular mechanisms (e.g., signal and volatiles, quorum sensing, chemical signal) of plant-microbe interactions and their potential in the phytoremediation process, which may contribute significantly to the practical application of phytoremediation techniques.

Although the impacts of PGPB on plant growth have been well investigated, limited information is available whether the application of PGPB promotes soil nutrient (e.g., N, P, and K) availability and the growth of fruit crops. A pot experiment containing four treatments was conducted by Shen et al. to evaluate the impacts of the complex PGPB inoculation on soil microflora, *Actinidia chinensis* growth, soil N fixation, and solubility of P and K. The results indicated an improvement of soil nutrient (N, P, and K) bioavailability, plant biomass, and N, P, and K uptake through the complex inoculant, suggesting this complex bacterial inoculant might be utilized as a biofertilizer for increasing soil fertility and thereafter plant growth.

Among PGPB, plant growth-promoting rhizobacteria (PGPR) are the essential component of phytoremediation technologies (Ma et al., 2011). To understand the roles of PGPR in microbe-aided phytoremediation, Fang et al. screened Cu/Zn-resistant PGPR isolates and assessed their bioremediation potential (plant growth enhancement and metal solubilization/tolerance/biosorption). Of those 10 Cu/Zn-resistant ACC-utilizing rhizobacterial isolates with superior PGP traits (e.g., P solubilization, production of IAA and siderophores), *Acinetobacter* sp. FQ-44 was chosen as the most profitable strain due to its abilities to 1) promote *Brassica napus* seedling growth under gnotobiotic conditions; 2) tolerate high concentrations of Cu and Zn; 3) mobilize the greatest amounts of water-soluble Cu, Zn, Pb, and Fe; and 4) adsorb the greatest amounts of Cu and Zn. The findings imply that *Acinetobacter* sp. FQ-44 might be exploited for microbe-aided phytoextraction. This study provided a viable method for screening metal-resistant PGPR that can be used to facilitate/accelerate phytoremediation of multi-metal contaminated soils.

In addition to plant growth promotion (e.g., production of ammonia, ACC deaminase, IAA and hydrogen cyanide) and metal uptake potential, Płociniczak et al. also studied the ability of PGPR *Brevibacterium casei* MH8a to colonize plant tissues using an antibiotic (e.g., rifampicin) as a biomarker. Furthermore, phospholipid fatty acid (PLFA) analysis was used to evaluate the ecological impacts of bioaugmentation on indigenous bacterial communities. The results demonstrated that the introduction of MH8a into soil resulted in its colonization of roots and leaves, enhanced *Salix alba* biomass and metal (Cd, Zn, and Cu) accumulation in roots, and temporary change in the structure of the autochthonous bacterial communities. The findings imply its long-term survival in soil, endophytic and PGP features, bioremediation potential, as well as its temporary impact on indigenous microbes.

Plants are often confronted simultaneously with both biotic and abiotic stresses, resulting in a reduction in plant growth and yield (Ma et al., 2016a). It is crucial to understand the impact of PGPR on multiple-stress amelioration and plant performance. Laksmanan et al. reported that plants subjected to arsenic (As) regime increased their susceptibility to infections of the blast pathogen *Magnaporthe oryzae*; however, inoculation of PGPR *Pantoea* sp. EA106 reduced blast infections and As uptake by rice. This is attributed to the up-regulation of defense-related genes mediated by *Pantoea* sp. EA106. The findings show the first evidence of how rice copes with mixed stress (As and blast infection) regimes.

Apart from the use of PGPR possessing biocontrol properties, developing resistant varieties *via* inserting resistance genes with marker-based breeding is an alternative to reduce and eliminate blast infections. Jain et al. used the resistant near-isogenic line (NIL) of Pusa Basmati-1(PB1) to investigate blast resistance gene *Pi9*-mediated resistance response. Moreover, they performed transcriptome profiling to unravel *Pi9*-mediated resistance mechanisms.

Besides PGPR, endophytic bacteria that colonize healthy plant tissues without causing symptoms have been receiving attention lately for their capacity to promote plant growth and thus phytoremediation. IAA produced by endophytic bacteria is known to play a crucial role in the interactions between plant hosts and their endomicrobes. Chen et al. evaluated the impacts of the bacterial endophyte *Pseudomonas fluorescens* Sasm05 and exogenous IAA on plant physiochemical traits, cadmium (Cd) uptake and the expression of metal transporter genes in *Sedum alfredii* Hance grown in a hydroponic media contaminated with Cd. The results demonstrated that both exogenous IAA and Sasm05 inoculation improved plant growth and photosynthesis; however, Sasm05 had a greater influence on uptake and translocation of Cd, suggesting that under Cd stress Sasm05 may produce IAA to stimulate plant growth and regulate the expression of Cd uptake and transport genes to improve plant Cd uptake.

The application of plant-endophyte symbiotic systems can also be a prospective approach to boost phytoremediation efficiency. Ma et al. (2016b) isolated, characterized and identified a multi-metal resistant and plant growth-promoting endophytic bacterium (PGPE) *Achromobacter piechaudii* E6S and evaluated its role in plant growth, metal uptake and translocation in *Sedum plumbizincicola*. Strain E6S was found to resist high concentrations of various metals (Cd, Zn, and Pb) and exhibit PGP traits (e.g., IAA production and P solubilization), increase soil metal (Cd, Zn, and Pb) bioavailability, and also bind considerable amounts of metal ions (Zn > Cd > Pb) on its cells. In the pot experiments, E6S inoculation enhanced plant biomass and accumulation of Cd, Zn, and Pb, but reduced translocation factor of Cd and Zn. The results indicate that *A. piechaudii* E6S can promote metal rhizoaccumulation and thus phytostabilization efficiency.

Similarly, Visioli et al. assessed the impacts of five specific nickel (Ni) resistant PGPE strains (individual and co-inoculation) on phytoextraction potential of Ni-hyperaccumulator *Noccaea caerulescens*. The results demonstrated that individual bacterial inoculation was not effective in increasing the growth and Ni translocation in *N. caerulescens*, except for *Arthrobacter* sp. Ncr-1 and *Microbacterium* sp. Ncr-8. Co-inoculation of Ncr-1 and Ncr-8 with *N. caerulescens* resulted in dense colonization of roots and leaf epidermal tissues and was more effective in the plant growth promotion, Ni removal from soil and translocation within the plant, together with that of Fe, Co, and Cu. The findings suggest that tolerance/adaptation of *N. caerulescens* to highly Ni-polluted serpentine soils can be improved by an integrated PGPE community.

Mesa et al. investigated that a high proportion of metal (As, Cu, and Zn) resistant endophytic bacterial strains were the most abundant in soil and tissues of metal bioaccumularor *Spartina maritima* growing in polluted estuaries. These strains possessed multi-enzymatic properties (e.g., amylase, cellulase, chitinase, protease, and lipase) and PGP properties (e.g., N fixation, P solubilization and production of IAA, siderophores, and ACC deaminase). After inoculating *S. maritima* with a consortium of PGPE strains, they found that endophytic inoculation increased plant photosynthesis and intrinsic water use efficiency, but reduced metal accumulation. The findings imply that inoculation of indigenous metal-resistant PGPE can be considered as a practical approach to facilitate/accelerate the adaption/tolerance and growth of *S. maritima* in polluted estuaries, but unsuitable for rhizoaccumulation purposes.

Air pollution results in adverse effects on human health and ecosystems. It is well known that plant leaves can absorb air pollutants, and leaf endophytes can transform contaminants into less or nontoxic forms; however, their integrated capacities for air remediation have been scarcely explored so far. Wei et al. reviewed bioremediation of air pollutants, with a focus on the advances in omics technologies and molecular basis underlying the role of plant leaves and leaf-associated microbes in the reduction of air pollutants, therefore providing theoretical bases for developing phylloremediation to mitigate pollutants in the air.

Besides indigenous microbes, engineering the plant-associated microbiome (PAM) is expected to promote plant survival, growth, and performance in contaminated soils. Yergeau et al. modified the PAM *via* gamma-irradiation followed by soil inoculation, which caused short-term shifts in microbial communities, but lasting impacts on growth traits of *Salix* sp. They hypothesized on the potential of manipulating the PAM to modify target plant characteristics. However, it did not occur in this study. They also highlighted several key factors when engineering the PAM.

Zhang et al. investigated flux profiles of Cd^2+^, Ca^2+^, and H^+^ in axenic cultures of two *Paxillus involutus* strains, ectomycorrhizal (EM) *Populus × canescens*, and non-mycorrhizal (NM) roots using a non-invasive micro-test technique. The results showed that EM *P. × canescens* roots maintained high production of H_2_O_2_ and activity of H^+^-pumping, which activated the plasma membrane Ca^2+^ channels and hence stimulated a high Cd^2+^ influx under Cd stress. They proposed a signaling pathway that triggered Ca^2+^ channel-mediated Cd^2+^ influx in the roots of NM *P.* × *canescens* and elucidated the indubitable Cd^2+^ stimulation in EM associations under Cd stress.

The potential ecological impacts of long-term growth of genetically modified plants (GMPs) have received increasing attention. Qi et al. examined fungal diversity associated with 15-year conventional cotton, 10-year *Bacillus thuringiensis* cotton, and 15-year transgenic cotton, and monitored the variation in fungal communities over an annual growth cycle. The results indicate that among different transgenic cultivars or lines variations of microbial diversity could exist, and the unintended variations between conventional and transgenic cotton may be within a generally acceptable range.

It is well known that bacterial ACC deaminase encoded by the gene *AcdS* is regulated differently under different environmental conditions and can help plants alleviate biotic and abiotic stresses by hydrolyzing ACC (a plant ethylene precursor) into ammonia and α-ketobutyrate, therefore reducing ethylene level and providing carbon and N for bacterial growth (Ma et al., 2016a, 2016b). Singh et al. explored current knowledge of bacterial ACC deaminase induced plant physiological changes, mechanism of enzyme action, genetics and distribution and its ecological role, as well as avenues for future research to explore transgenic plants expressing a foreign *AcdS* gene to adapt to environmental stresses.

Most terrestrial plant roots form a symbiosis with AMF, which contributes significantly to nutrient cycling and ecosystem sustainability. To understand the role of AMF in nutrient acquisition and soil stress alleviation, Aliferis et al. attempted to elucidate the changes in the metabolic response of *Salix purpurea* during AMF symbiosis through recording the fluctuation of leaf metabolome. The results revealed that AMF inoculation caused up-regulation of various biosynthetic pathways (e.g., flavonoid, isoflavonoid, phenylpropanoid, chlorophyll, and porphyrin), which had important roles in plant physiology and resistance to various environmental stresses. The fluctuation in leaf metabolism may provide AMF-inoculated *S. purpurea* with a significant advantage when grown in highly polluted soils. The discovered biomarkers of *S. purpurea* response to AMF inoculation and corresponding pathways might be utilized in the biomarker-based selection of *S. purpurea* cultivars with high phytoremediation potential.

Besides PGPB and AMF, certain filamentous fungi (e.g., *Trichoderma* sp.) have great potential to improve plant establishment and thus phytoremediation capacity (Bareen et al., 2012). Teng et al. explored the impacts of Cd-tolerant *Trichoderma reesei* FS10-C on soil fertility and phytoremediation of Cd-polluted soil by *S. plumbizincicola*. The results showed that two inoculation agents containing FS10-C were better on all accounts compared to those without FS10-C. Moreover, solid fermentation powder was proposed as an efficient inoculation agent for FS10-C to improve soil fertility and Cd phytoremediation, as it had the greatest potential to promote plant growth, Cd accumulation, nutrient availability, as well as microbial biomass and activities.

Scientists from basic science to applied science are contributing significantly to a better understanding of physicochemical, molecular, and cellular mechanisms involved in plant-microbe-metal interactions under various abiotic and biotic stresses, which will certainly help develop novel solutions for PGPM-aided phytoremediation and restoration strategies.

## Author Contributions

YM drafted the editorial text, revised and approved the final version of the editorial text.

## Conflict of Interest

The author declares that the research was conducted in the absence of any commercial or financial relationships that could be construed as a potential conflict of interest.
